# RNA editing increases the nucleotide diversity of SARS-CoV-2 in human host cells

**DOI:** 10.1371/journal.pgen.1010130

**Published:** 2022-03-30

**Authors:** Xinxin Peng, Yikai Luo, Hongyue Li, Xuejiao Guo, Hu Chen, Xuwo Ji, Han Liang

**Affiliations:** 1 Precision Scientific (Beijing) Co., Ltd., Beijing, China; 2 Department of Bioinformatics and Computational Biology, The University of Texas MD Anderson Cancer Center, Houston, Texas, United States of America; 3 Graduate Program in Quantitative and Computational Biosciences, Baylor College of Medicine, Houston, Texas, United States of America; 4 Department of Systems Biology, The University of Texas MD Anderson Cancer Center, Houston, Texas, United States of America; ISPRO, ITALY

## Abstract

SARS-CoV-2 is a positive-sense, single-stranded RNA virus responsible for the COVID-19 pandemic. It remains unclear whether and to what extent the virus in human host cells undergoes RNA editing, a major RNA modification mechanism. Here we perform a robust bioinformatic analysis of metatranscriptomic data from multiple bronchoalveolar lavage fluid samples of COVID-19 patients, revealing an appreciable number of A-to-I RNA editing candidate sites in SARS-CoV-2. We confirm the enrichment of A-to-I RNA editing signals at these candidate sites through evaluating four characteristics specific to RNA editing: the inferred RNA editing sites exhibit (i) stronger ADAR1 binding affinity predicted by a deep-learning model built from ADAR1 CLIP-seq data, (ii) decreased editing levels in ADAR1-inhibited human lung cells, (iii) local clustering patterns, and (iv) higher RNA secondary structure propensity. Our results have critical implications in understanding the evolution of SARS-CoV-2 as well as in COVID-19 research, such as phylogenetic analysis and vaccine development.

## Author summary

The COVID-19 pandemic is caused by SARS-CoV-2, an RNA virus. In the cells of COVID-19 patients, SARS-CoV-2 interacts with human proteins and is potentially subjected to their enzymatic activities. Here we investigated whether human protein enzymes can change the nucleotide sequence of SARS-CoV-2, thereby leaving a unique molecular footprint. We developed a robust computational algorithm to analyze the sequence data of SARS-CoV-2 obtained from lung fluid samples of COVID-19 patients and found that the virus contains new nucleotide changes that are likely induced by ADAR1, a powerful human protein that can modify specific nucleotide positions in many human transcripts. We further confirmed that the characteristics of the nucleotide changes detected in SARS-CoV-2 are similar to those observed in the human genes. Thus, these ADAR1-induced nucleotide changes may represent an under-appreciated force that can affect the evolution of SARS-CoV-2. Our study helps researchers better understand the evolutionary trajectory of SARS-CoV-2.

## Introduction

The rapid spread of coronavirus disease 2019 (COVID-19) across the world represents an urgent healthcare emergency. By January 2022, the virus had infected >352 million people and caused >5.6 million deaths globally, and these numbers continue to increase. COVID-19 is caused by a novel coronavirus designated as severe acute respiratory syndrome coronavirus 2 (SARS-CoV-2) [1,2]. In the past two years, extensive efforts have been made to characterize this highly contagious virus: the genomes from thousands of infected patients have been sequenced, and the transcriptome architecture has been determined. The genome of SARS-CoV-2 is a positive-sense, single-stranded RNA of ~30 kb and contains ten canonical RNA products in addition to a few unknown ORFs [1–3]. These results have provided a key foundation for elucidating the evolutionary pattern and pathogenicity of SARS-CoV-2 and for developing effective treatment strategies. However, our knowledge of nucleotide variation and plasticity of this viral genome is still limited, especially RNA modifications induced in human host cells.

**Fig 1:**
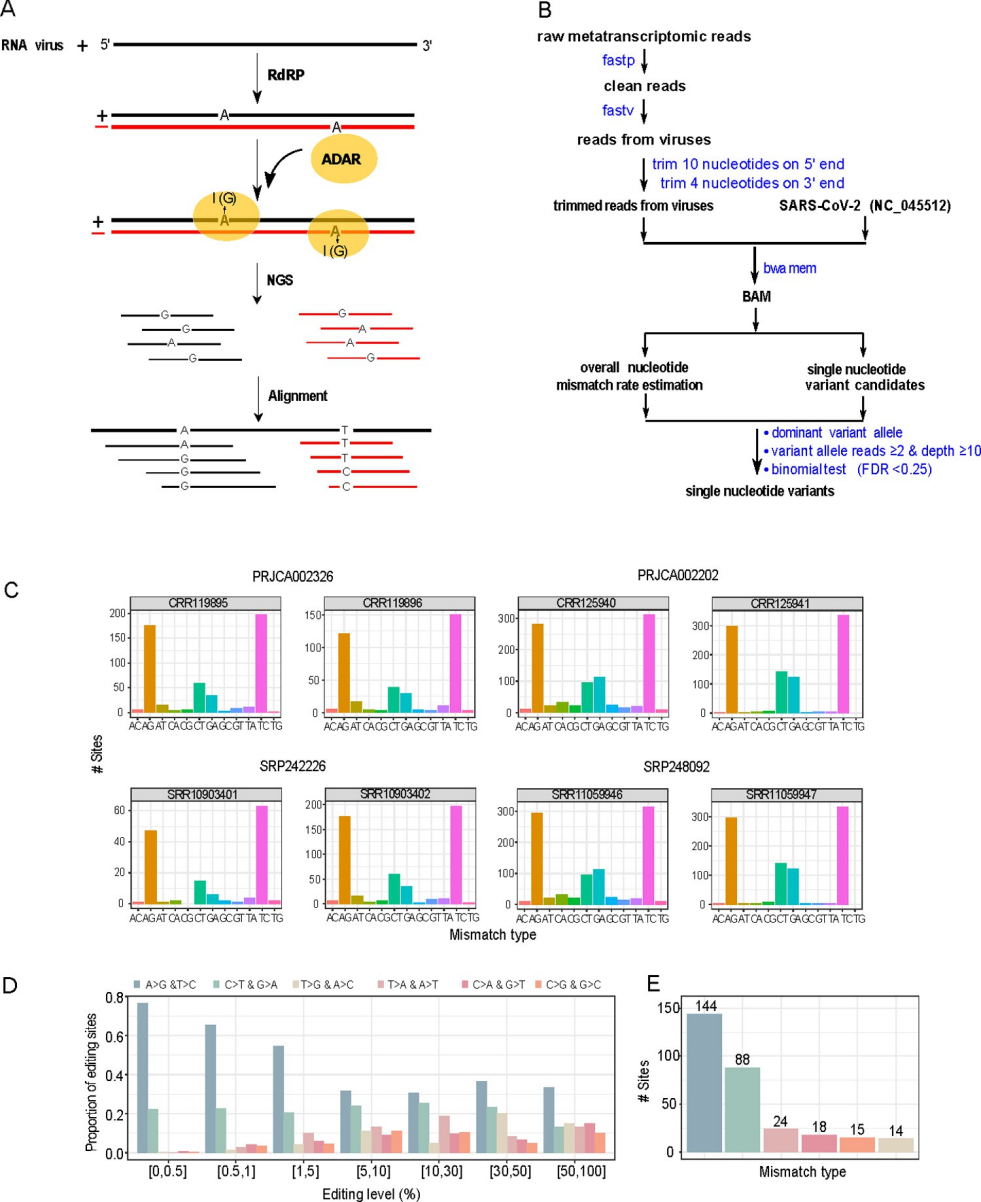
Identification of A-to-I RNA editing candidate sites from metatranscriptomic sequencing data from COVID-19 patient samples. (A) A cartoon illustration showing ADAR-mediated A-to-I RNA editing on positive and negative strands of SARS-CoV-2, which causes A>G and T>C substitutions, respectively. (B) The bioinformatics pipeline for identifying SNVs. (C) The distribution of 12 possible SNV types in eight representative samples (two per study). See the remaining samples investigated in S2 Fig. (D) The distribution of six distinct SNV types at different VAF cutoffs (paired SNV types corresponding to the same SNV changes in positive and negative strands are combined). (E) The distribution of six distinct SNV types was identified with the same procedure (VAF: 0.5%-70%, and recurrence ≥ 3 samples).

RNA editing is a widespread nucleotide modification mechanism through which specific nucleotides are modified by RNA editing enzymes at the RNA level without altering template genomic DNA [4]. Adenosine to inosine (A-to-I) is the most prevalent editing type in humans [5]. The A-to-I conversion is catalyzed by adenosine deaminases that act on RNA (ADARs), and the resulting inosines are recognized as G by the translational machinery [6,7]. The other known RNA editing type is cytidine to uridine (C-to-U), which is catalyzed by APOBEC1 [8]. Upon entering human cells, whether and to what extent SARS-CoV-2 is subjected to the activities of human RNA editing enzymes remains largely unexplored. This knowledge is of importance for at least two reasons. First, as the virus employs its negative-strand RNA as a replication template [9] (**Fig 1A** shows the example of A-to-I RNA editing), the nucleotide changes thus induced could become a direct source of genetic variations inherited from generation to generation. Second, in sharp contrast to the human genome, the vast majority of the SARS-CoV-2 genome is protein-coding, and thus, RNA editing events would have a much higher probability of causing amino acid changes, thereby modifying protein products. Although identifying RNA editing events from RNA-sequencing data has been well described in many species, including humans, such an analysis for an RNA virus is not trivial. This is because, without the DNA sequence for comparison, it is almost impossible to distinguish single nucleotide variants (SNVs) caused by spontaneous mutation processes from those due to RNA editing, solely based on alignment-based sequence analysis. In this study, our strategy was to first identify a high-confidence nucleotide variant candidate pool from metatranscriptomic sequencing reads of COVID-19 patient samples using a robust bioinformatics pipeline and then test whether real RNA editing signals were enriched in the candidate pool. To do so, we evaluated multiple RNA editing-specific characteristics of the candidate sites in comparison to other A/T sites in the SARS-CoV-2 genome, including (i) ADAR1 binding affinity predicted by a deep-learning model based on ADAR1 CLIP-seq data; (ii) cause-effect relationship between ADAR1 expression and the global RNA editing level based on a drug-treated human cell line perturbation experiment, (iii) local clustering patterns of candidate sites from a distance-based analysis, and (iv) RNA secondary structure propensity. The results from these analyses strongly suggest that an appreciable proportion of the RNA variants we identified result from the ADAR1-mediated A-to-I RNA editing process.

## Results

**Fig 2:**
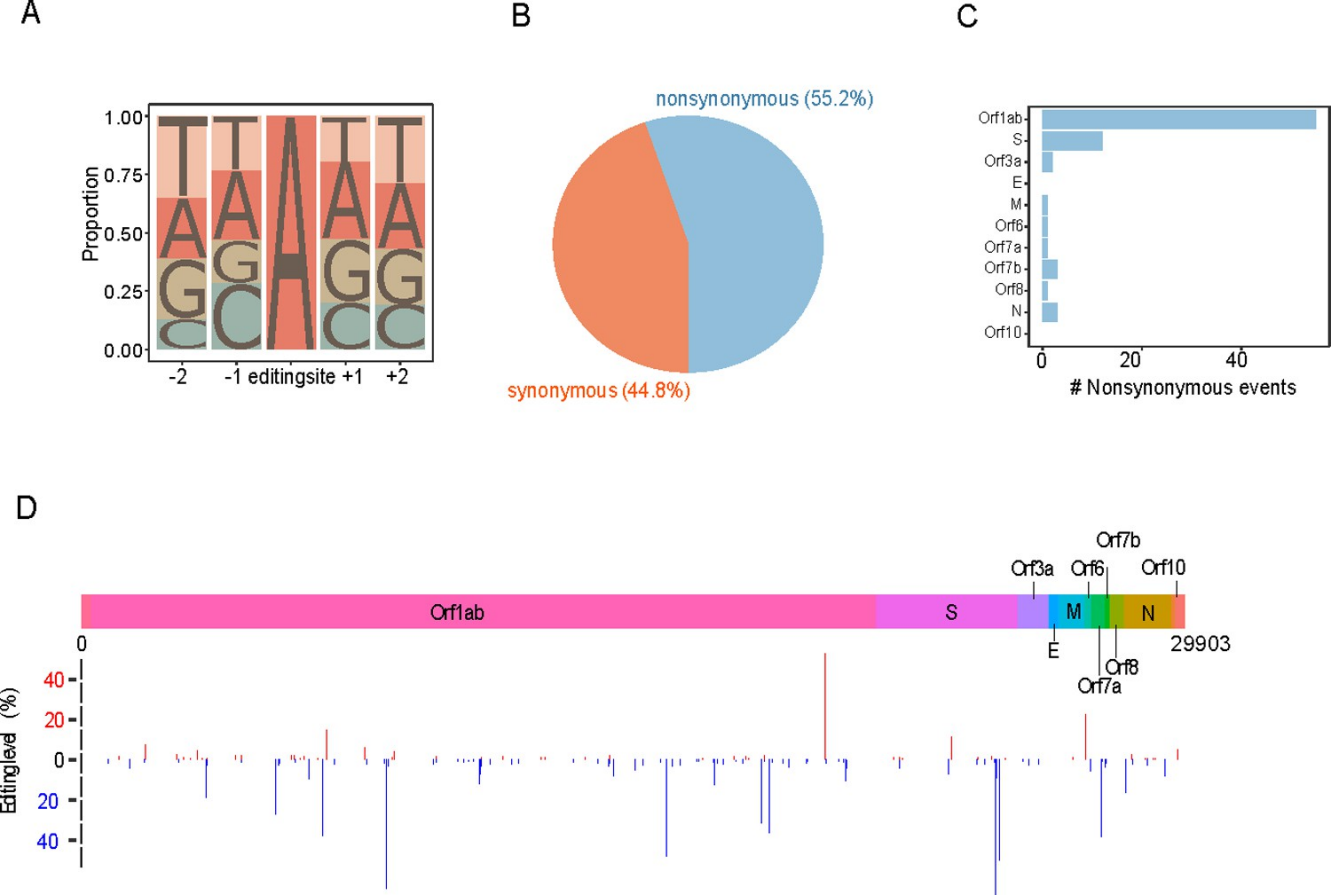
Functional impact and genomic distribution of A-to-I RNA editing candidate events. (A) Sequence context of the inferred RNA editing sites. (B) Pie chart showing the proportion of nonsynonymous and synonymous RNA editing sites; (C) bar chart showing the numbers of nonsynonymous editing events in different annotated genes; and (D) the distribution of RNA editing events along the SARS-CoV-2 genome. A>G and T>C sites are colored red and blue, respectively, and the height indicates the editing level (VAF). S, spike protein; E, envelope protein; M, membrane protein; and N, nucleocapsid protein.

To study the potential effects of A-to-I RNA editing in SARS-CoV-2, we first performed a systematic analysis of metatranscriptomic sequencing reads of bronchoalveolar lavage fluid samples of COVID-19 patients obtained from four independent studies (**S1 Table**). We developed a rigorous bioinformatics pipeline to detect SNVs, which includes (i) removing low-quality reads, (ii) identifying viral reads using Fastv [10], (iii) trimming end nucleotides due to their higher error rates, (iv) generating high-quality alignment, and (v) detecting SNVs with a significant variant allele frequency (VAF) above the background mismatch rate (**Fig 1B**). For 19 samples investigated, one sample had no detectable SNVs; and among the remaining samples, we observed a consistent pattern of A>G and T>C substitutions showing the highest abundance, followed by C>T and G>A substitutions in 17 samples (**Figs 1C, S1, and S2**). The dominance of the two SNV types, A>G and T>C, corresponding to potential A-to-I RNA editing events (in the positive and negative strands, respectively), was consistent at different VAF cutoffs across the 17 samples (**Fig 1D**). It should be emphasized that several sources may contribute to these observed SNVs, including sequencing errors, single nucleotide polymorphisms (SNPs, the fixed nucleotide differences between the studied virus and the reference virus genome), *de novo* mutations, and acquired RNA editing events in human cells. To distinguish high-confidence A-to-I RNA editing events from other types of variations, we applied a series of filters to the A>G/T>C variants in the 17 samples. First, given (i) the Illumina sequencing error rate is known to be ~0.1% [11] and (ii) the SARS-CoV-2 mutation rate is estimated to be similar to that of the mouse hepatitis virus (MHV), which is 2.5×10^−6^ substitutions per site per cell infection [12], we filtered those with VAF < 0.5% to remove the potential contaminations of sequencing errors and *de novo* mutations as well as very weak RNA editing sites. Second, given the prevalence of SARS-CoV-2 SNPs has been estimated to be 9.6 nucleotides between any two viral sequences [13], we also excluded a handful of SNVs with VAF > 70% since they are likely to be of such a source. Third, we focused on those recurrent editing sites in at least 3 out of the 17 samples. In total, we identified 144 recurrent A>G/T>C SNVs with VAF of 0.5–70% to obtain a high-confidence set of A-to-I RNA editing candidates, which is far more than any other SNV types based on the same procedure (**Fig 1E** and **S2 Table**). We further examined the flanking sequences of these candidate editing sites and observed a preference for G depletion and enrichment at the nucleotides 5’ and 3’ to the editing sites (-1 and +1 position), respectively, which is consistent with the context signal previously reported in human transcripts (**Fig 2A**) [14–16]. In terms of functional impact, 55% of the editing sites would cause nonsynonymous substitutions (**Fig 2B**), most of which are in ORF1ab, followed by the spike protein (**Fig 2C**). **Fig 2D** shows their position distribution along the viral RNA genome.

**Fig 3:**
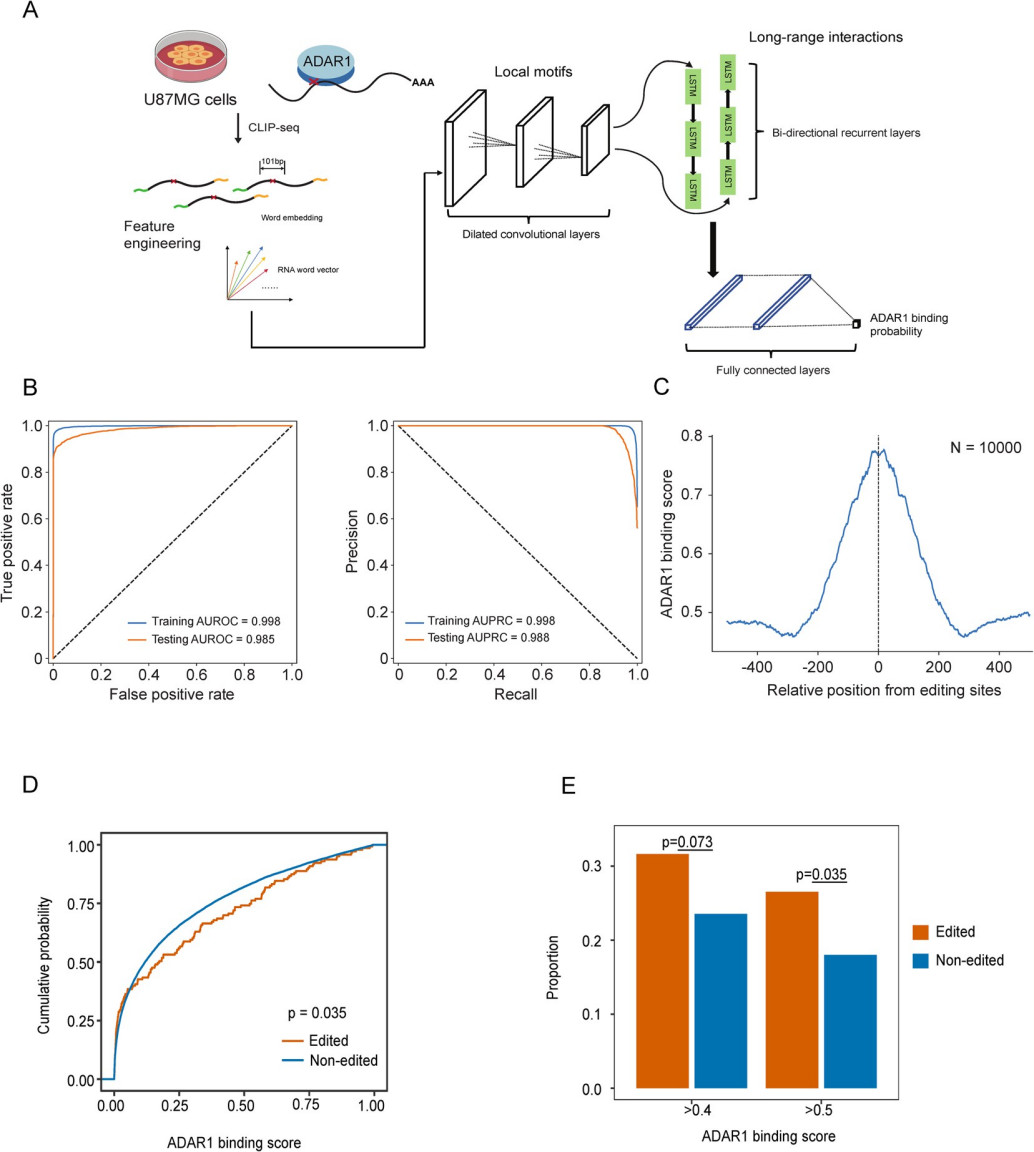
ADAR1 binding affinity at inferred A-to-I RNA editing candidate sites. (A) A cartoon illustration of the deep-learning model for sequence-based ADAR1 binding affinity prediction. (B) Performance evaluation of the prediction model based on cross-validation. (C) The distribution of ADAR1 binding affinity scores centered on 10,000 known A-to-I RNA editing sites in the human genome. (D) Empirical cumulative distribution of ADAR1 binding scores for the inferred RNA editing sites and other A/T positions in the SARS-CoV-2 genome. P-value: one-sided Kolmogorov-Smirnov test. (E) Enrichment of RNA editing sites in high ADAR1 affinity score groups. A/T sites in the SARS-CoV-2 genome without editing signals were used as the background for comparison. P-value: Chi-square test.

Although we followed the best common practice in the RNA-editing field to identify a set of high-confidence A>G/T>C mismatch positions that served as an A-to-I RNA editing candidate pool, as discussed above, it is impossible to exclude the potential contributions of other sources, e.g., *de novo* mutations. To address this challenge, we sought to test whether our candidate pool was enriched for genomic and functional features that are known to be specific to A-to-I RNA editing. ADAR1 is the major enzyme responsible for most A-to-I RNA editing signals observed in humans [17,18]. Because an RNA virus replicates in the cytoplasm and the human p150 isoform of ADAR1 is present in this compartment as well, it is supposedly the major factor responsible for viral A-to-I RNA editing activity [19]. Thus, we reasoned that if a large proportion of the inferred RNA editing sites in SARS-CoV-2 are authentic, these sites would be expected to have higher ADAR1 binding affinity than other A/T sites, which can be evaluated through a sequence-based binding affinity prediction model. Based on a recent ADAR1 CLIP-seq peak set [20], we built a hybrid neural network consisting of a dilated deep convolutional neural network, a deep recurrent neural network, and eventually, a fully-connected layer inspired by the deepRAM architecture [21], which is designed to effectively capture the RNA sequence context around ADAR1 binding peaks (including both local motifs, and long-range interactions) (**Fig 3A**). We achieved extremely high performance with this model in cross-validation (training set, area under receiver operating characteristic curve [AUROC] = 0.998 and area under precision-recall curve [AUPRC] = 0.998; testing set, AUROC = 0.985 and AUPRC = 0.988, **Fig 3B**). We further validated the model performance using 10,000 independent human A-to-I RNA editing sites [22] and observed a sharp peak with a fairly low variance of ADAR1 binding scores centered on these known sites, further supporting the high accuracy of our model (**Fig 3C**). Notably, because our model was trained based on human sequences, which inevitably caused the model to learn features specific to both ADAR1 binding and the human genomic context, predicted ADAR1 binding affinity scores cannot be compared across different species directly. Instead, it is more appropriate to compare different sites for their relative ADAR1 binding affinity within the same species because they share the same genomic context. Confirming our hypothesis, the RNA editing sites detected in SARS-CoV-2 showed a significant shift towards higher ADAR1 binding scores (Kolmogorov-Smirnov test, p = 0.035, **Fig 3D**). We also found that the enrichment ratio increased with the score cutoff value (**Fig 3E**). This result indicates that many of these RNA editing candidate sites indeed tend to bind to ADAR1.

**Fig 4:**
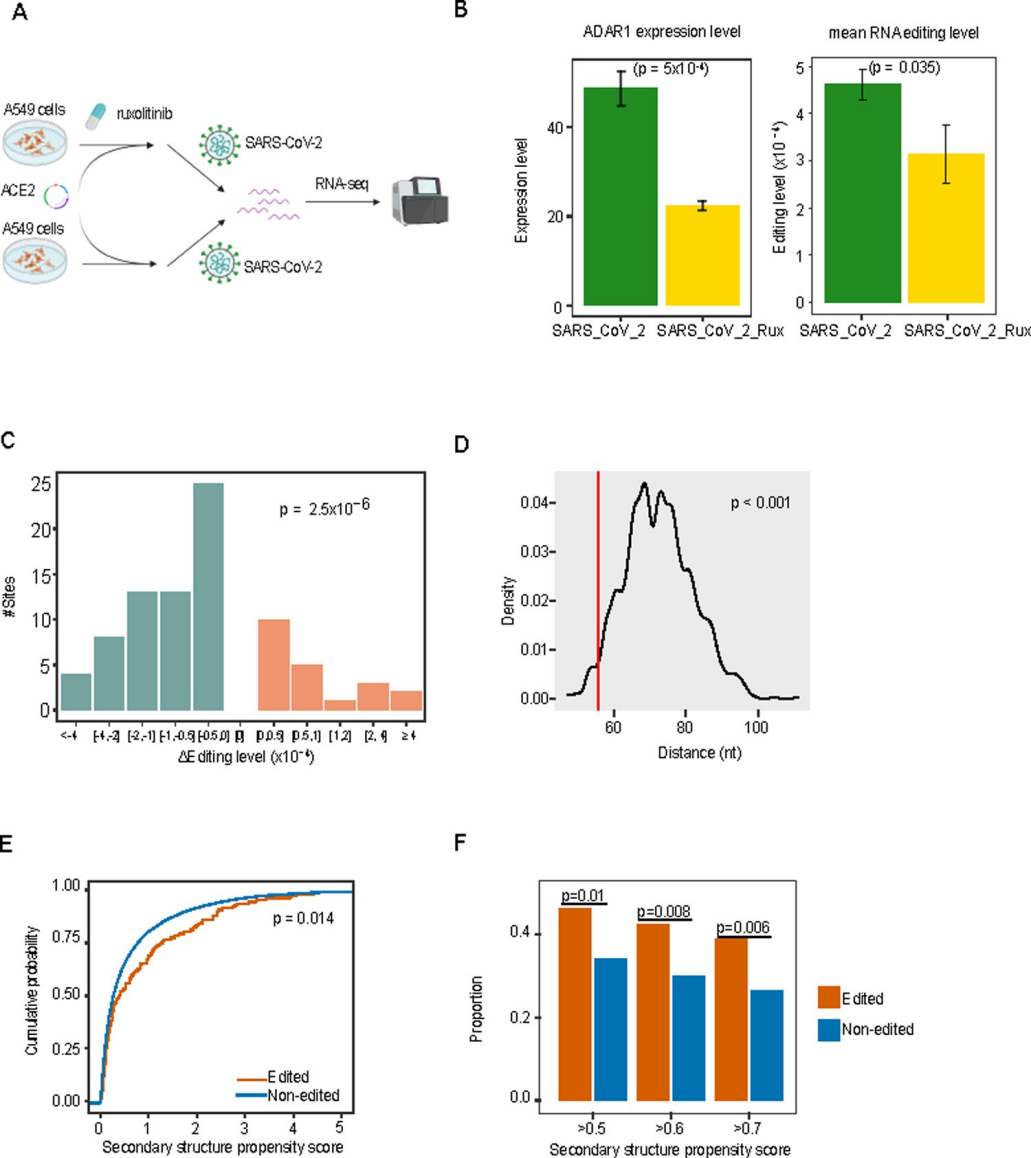
Further evidence supporting inferred A-to-I RNA editing sites. (A) The flowchart of the cell line perturbation experiment (three replicates in each group). (B) Left, the expression levels of ADAR1 in the SARS-CoV-2-infected cell lines with and without ruxolitinib treatment. Right, the RNA editing levels in the two groups. The mean values across the RNA editing sites were used for comparison. P-value: Student’s t-test. (C) The distribution of the editing-level change at the 84 RNA editing sites with sufficient coverage (≥×10) and showing a varied editing level upon the drug treatment. P-value: one-sided binomial test. (D) The clustering pattern of RNA editing sites in the SARS-CoV-2 genome. Given a set of RNA editing sites, the distance of each site to its nearest neighbor was calculated, and the median value across different sites was used as an index. The distribution of median distance values was obtained from 1,000 random sets, and the vertical line indicates the value from the true RNA editing set. P-value: permutation test. (E) Distribution of SARS-CoV-2 secondary structure propensity scores between the RNA editing candidate pool and otherwise. P-value: Kolmogorov-Smirnov test. (F) Enrichment of RNA editing sites in high secondary structure propensity score groups.

We further evaluated other RNA-editing specific features of the candidate pool in multiple aspects. First, to test the causal relationship between the expression of ADAR1 and the global RNA editing level, we obtained the RNA-seq data generated from a human lung cell line model following SARS-CoV-2 infection (**Fig 4A**) [23]. In the infected human cells, the ADAR1 expression level was significantly inhibited by an immunosuppressive reagent, ruxolitinib (t-test, p = 5×10^−4^, **Fig 4B;** the inhibitory effect was more striking for p110 mRNA isoform, **S3 Fig**). Consistently, we observed much lower average RNA editing levels across the candidate sites (t-test, p = 0.035, **Fig 4B**). In addition to the sample-wise comparison, we analyzed the editing-level change per site and found that 63 out of the 84 (75%) editing sites with sufficient coverage showed a decreased editing level, significantly higher than random expectation (one-sided binomial test, p = 2.5×10^−6^, **Fig 4C**). This result demonstrated a direct effect of host ADAR1 on the dynamics of viral A-to-I RNA editing. Second, RNA editing sites are known to form local clusters. Indeed, the RNA editing candidate sites showed a much shorter distance to neighboring RNA editing candidate sites than randomly sampled, same-size A/T control sets (permutation test, p < 1×10^−3^, **Fig 4D**). Third, A-to-I RNA editing is known to be specific to double-stranded RNA structures. Using a computational RNA structure prediction algorithm, CROSS [24], we assessed the secondary structure propensity of the SARS-CoV-2 sequence and found that the inferred RNA editing sites were enriched in regions with significantly higher RNA secondary structure propensity (Kolmogorov-Smirnov test, p = 0.014, **Fig 4E**). Indeed, the proportion of RNA editing sites was significantly higher than that of non-edited sites in secondary structure regions using different propensity-score cutoffs (**Fig 4F**). These multiple lines of evidence strongly suggest that a considerable proportion of our inferred RNA editing candidate sites result from ADAR1-mediated A-to-I RNA editing.

**Fig 5:**
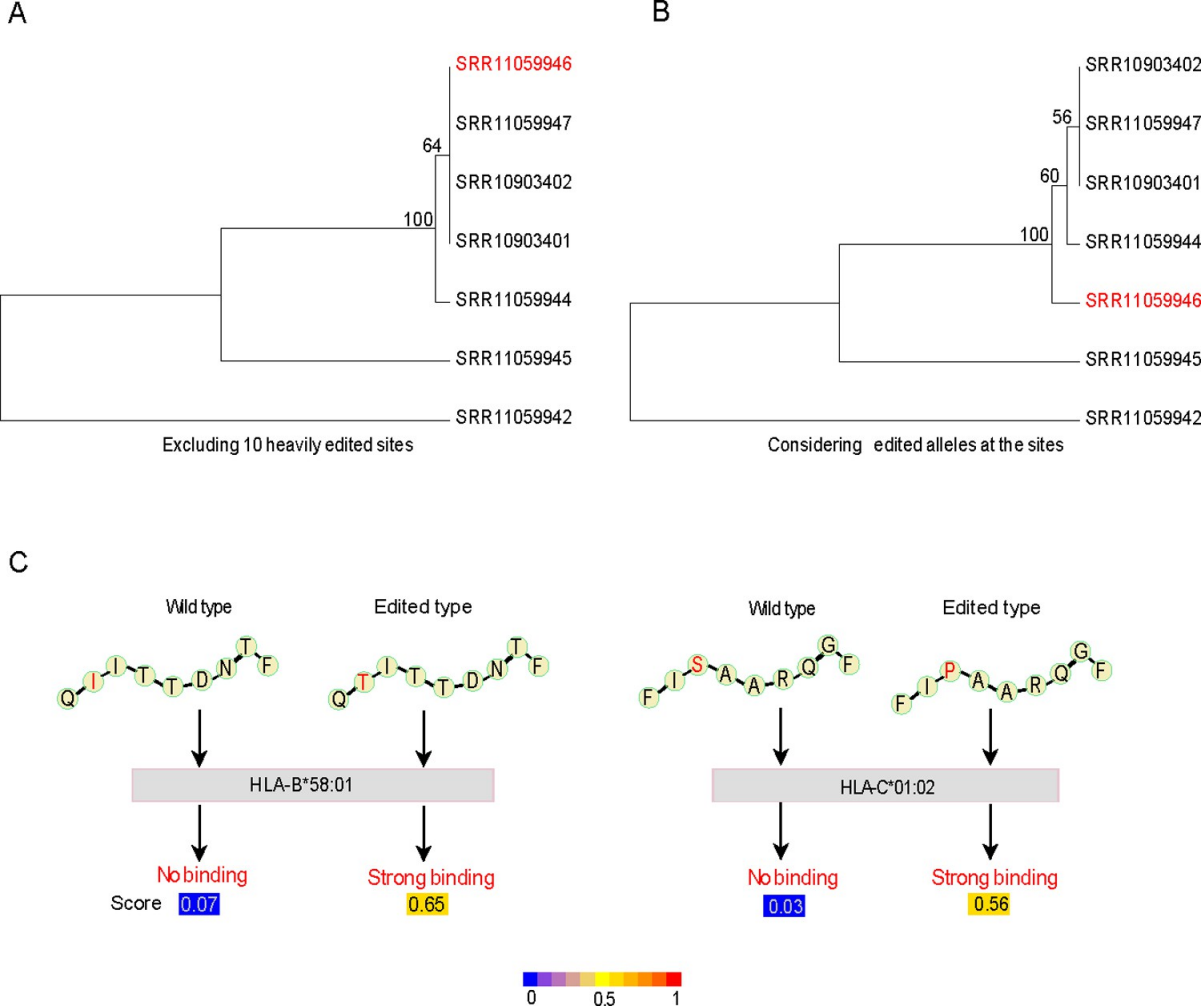
Potential effects of RNA editing on COVID-19 research. (A, B) Phylogenetic topology based on SARS-CoV-2 genomes without (A) and with (B) considering the 10 heavily edited sites. Bootstrap values are shown on the internal nodes. A sample showing the cluster shift is highlighted in red. (C) Two representative examples show that the amino acid changes caused by A-to-I RNA editing potentially increase the binding affinity of epitopes to HLA.

Finally, we examined the potential impact of A-to-I RNA editing events on two aspects of COVID-19 research. First, the phylogenetic analysis of SARS-CoV-2 plays a key role in studying the virus origin and evolutionary patterns. Although the vast majority of the RNA editing events have a low editing level, several cases can reach a very high level (e.g., ≥ 30%), thereby likely being identified as major alleles in the genome assembly. Thus, RNA editing signals may confound the phylogenetic inference. To demonstrate this point, we compared phylogenetic trees for seven samples from two studies (SRP142226 and SRP248092) after either excluding the 10 heavily edited sites (**Fig 5A**) or considering the edited alleles at these sites (**Fig 5B**) and found distinct tree topologies. Second, epitope-based vaccines have been under intensive investigation for COVID-19 prevention. We recently reported that RNA editing contributes to peptide diversity, and editing-derived epitopes can elicit immune responses in cancer cells [25,26]. Focusing on recurrent RNA editing events across samples, we assessed the effects of RNA-editing-induced amino acid changes on the binding affinity of the T-cell epitope to HLA and found a few cases where the edited peptide significantly increased the binding affinity relative to the wild-type peptide (**Fig 5C and S3 Table**).

## Discussion

In this study, we provide global evidence that SARS-CoV-2 undergoes ADAR-mediated A-to-I RNA editing in human cells. Although it remains unclear as to what extent the detected RNA editing occurs in the virus genome vs. transcribed RNA products, besides spontaneous mutations, RNA editing may represent another source of genetic variants that can shape the plasticity and evolution of this virus. SARS-CoV-2 genome replication mainly takes place in the cytoplasm. Besides the ADAR1 p150 isoform, which is present in the cytoplasm, our results suggest that the ADAR1 p110 isoform plays a role in the RNA editing activity for SARS-CoV-2, which is consistent with a recent study showing that p110 acts as a restriction factor for influenza virus [19]. In sum, the RNA editing events identified across the virus genome are likely mediated by ADAR1, as supported by our assessments on ADAR1 binding affinity and cause-effect pattern of ADAR1 expression and RNA editing activities.

Our study has several limitations. First, although we observed consistent A-to-I RNA-editing signals above the background, the signal-to-noise of our RNA editing calls is not high. This is mainly due to two reasons: (i) the A>G/T>C mismatches are more enriched among SNVs with an extremely low VAF (Fig 1D), but we only included those variants reaching a significant editing level to focus on the RNA editing events with a meaningful biological impact; (ii) because of the distinct sequence context of SARS-CoV-2, the ADAR1 binding model built from human sequence data may be under power to distinguish true RNA editing sites from the background noise. Additional efforts should be made to detect RNA editing events using a more accurate bioinformatic pipeline. Second, our study did not include direct experimental validation for the inferred RNA editing sites. For example, an assessment of the effects of ADAR1 inactivation on RNA editing patterns would provide more convincing evidence.

As an additional source of genetic variations, A-to-I RNA editing induced by human host cells would accelerate the overall evolution of SARS-CoV-2. Similar to spontaneous mutations, the fate of an RNA editing event depends on the fitness effect it causes: it can be subjected to purifying selection if it is deleterious or positive selection if it is advantageous. Given the very low editing levels, the fixation probability of the vast majority of A-to-I RNA editing events would probably be low. However, quantitative assessment of the fixation and evolutionary fate of such RNA editing events is challenging due to several reasons. First, it is hard to estimate the real RNA editing rate per generation from the observed VAF in bulk RNA-seq data, as the RNA editing level can be a result of multiple generations (due to multiple replications in a host cell, multiple cell infections within an individual human host, or even multiple hosts). Second, our knowledge about the fitness landscape of RNA editing events in SARS-CoV-2 is very limited (if any). Third, it remains unclear which model the virus evolution follows in host cells (e.g., “explosive growth” vs. “equilibrium”). Interestingly, we also observed C>T/G>A peaks in the SNV spectrum, which might reflect C-to-U RNA editing. These two types of RNA editing processes may cancel each other’s effects on GC content in the viral genome to some extent. Further efforts are required to investigate the functional consequences of A-to-I editing events and assess whether C-to-U RNA editing also exists.

We note that two recent studies reported similar host-dependent RNA editing activities of SARS-CoV-2 in human cells [27,28]. However, we would like to emphasize three novel aspects of our study. First, through multiple independent analyses, including a deep-learning-based ADAR1 binding affinity model, we provide more convincing evidence for the inferred A-to-I RNA editing of SARS-CoV-2, which is independent of the alignment-based SNV profile analysis. Second, we show that the nucleotide variations induced by RNA editing could confound phylogenetic analysis, a key approach to inferring the evolutionary origin of SARS-CoV-2. Third, our results suggest that RNA-editing-derived peptides may serve as epitopes for vaccine development. However, A-to-I RNA editing has been considered as one of the mechanisms to suppress the innate immune response induced by dsRNA in human cells [29,30] and has also been shown to be used by RNA viruses to affect immune evasion [31,32]. Thus, neoantigens due to RNA editing events may only represent a limited adverse effect in the interactions between the virus and host cells. Together, our study provides critical insights into the evolution of SARS-CoV-2 and highlights a need to consider these host-induced nucleotide variants in future COVID-19 research.

## Materials and methods

### Sequencing data and preprocessing

All the sequencing data were generated as metatranscriptomic reads from bronchoalveolar lavage fluid of COVID-19 patients. We employed Fastp [10] to obtain clean reads and then Fastv (https://github.com/OpenGene/fastv) to extract viral reads.

### Single nucleotide variant detection

Viral reads were aligned against the reference genome of SARS-CoV-2 (positive virus strand, NC_045512.2) with BWA MEM [33]. We first estimated the number of mismatches at different nucleotide positions in the reads. To do so, we mapped clean reads that passed quality control to the reference genome and calculated the mismatch frequencies at both read ends and observed that the first ten and the last four nucleotides (from 5’ end) showed relatively high mismatches, suggesting higher sequencing error rates in these positions. We, therefore, trimmed these nucleotides from each clean read and realigned the reads. We focused on 19 samples with ≥ 20,000 clean reads mapped to the SARS-CoV-2 genome for downstream analysis. For each position with alternative allele(s) relative to the reference genome, we focused on the positions (depth ≥ 10) with a dominant alternative allele, which was defined as # reads of the dominant alternative allele > 10 × # reads of the remaining alternative alleles (if any). To further exclude SNVs likely caused by sequencing errors, we first empirically estimated the overall mismatch rate for each sample, followed by a binomial test, and only kept SNVs with a dominant alternative allele showing FDR < 0.25 and supported by at least two reads. Among the 19 samples, one sample had no detectable SNVs, and another sample did not show the A>G/T>C enrichment. We, therefore, focused on the remaining 17 samples for further analyses. To identify high-confidence A-to-I RNA editing events (A>G/T>C), we first retained those RNA editing events with an editing level of 0.5–70% in each sample and then selected those sites with a recurrence in ≥ 3 samples. We repeated the same procedures to identify other SNV types for comparison. We employed ANNOVAR [34] to annotate 144 unique RNA editing sites based on the gff file for the SARS-CoV-2 genome (https://www.ncbi.nlm.nih.gov/nuccore/NC_045512.2/). We extracted the flanking sequences centered on each editing site to assess the preferred sequence contexts.

### Construction and validation of an ADAR1 binding affinity prediction model

We employed a state-of-the-art deep neural network architecture as detailed previously [21] to build a prediction model that can evaluate the binding affinity of the human ADAR1 protein to SARS-CoV-2 genomic sequences. Briefly, the deepRAM architecture was based on a hybrid of a dilated deep convolutional neural network (CNN) and a deep recurrent neural network (RNN) to fully take advantage of the rich information embedded in the RNA sequence context (including both local motifs and long-range interactions). An automatic model parameter-sweeping procedure was used to ensure a parameter set that optimized the model performance. To construct positive input data sets, we randomly extracted 20,000 101-bp RNA sequences centered on the peak summit from an ADAR1 binding peak set generated from a CLIP-seq experiment in the human U87MG cell line [20]. We built negative sets by applying dinucleotide-frequency-preserving-shuffling to the positive sets to discourage the model from discriminating foreground sets from background sets by low-level genomic features only, such as GC content [35]. We randomly divided our data into 80% and 20% for training and testing, respectively. Following word2vec transformation, sequence features were propagated through CNN, RNN, and eventually a fully connected layer, where a sigmoid function was used to bound the network output in between 0 and 1, representing the binding probability (**Fig 2A**). In light of a 40-round hyper-parameter random calibration, we ended up with a model with a CNN layer of 32 filters, a bi-LSTM layer of hidden size 100, an Adagrad optimizer, a Xavier initializer, a learning rate of 0.046, a dropout ratio of 0.3, and the number of learning steps as 5,000.

To rigorously validate the ability of our prediction model to identify true A-to-I RNA editing sites, we assessed whether it would robustly distinguish the proximal flanking sequences of known RNA editing sites from the distal ones. We first randomly selected 10,000 RNA A-to-I editing sites from a pool of RNA events in a lymphoblastoid cell line annotated in the RADAR database [22]. Then, we partitioned the 1,101-bp region centered on the RNA editing sites into consecutive 1,001 101-bp windows with a step size of 1 bp. Finally, we scanned these windows with our model to generate a continuous ADAR1 affinity distribution. We compared the ADAR1 binding scores between the 144 RNA editing sites and those sites without editing signals detected in any sample.

### Analysis of RNA editing in the drug-treated cell line perturbation experiment

To validate whether the identified RNA editing sites are directly modulated by ADAR1 activity, we analyzed a public RNA-seq dataset in which ADAR1 was inhibited (three replicates in the drug-treated and control groups, GSE147507, series 16). In brief, the SARS-CoV-2 receptor ACE2 was over-expressed in a lung adenocarcinoma cell line, A549. The cells were then treated with ruxolitinib (a JAK1 and 2 kinase inhibitor) or control, denoted as SARS-CoV-2_Rux and SARS-CoV-2, respectively, and infected with SARS-CoV-2. The expression level of ADAR1 and its isoforms was calculated by Cufflinks, and a two-tailed Student’s t-test was employed to evaluate the statistical significance between the two groups. To quantify RNA editing level across the six samples, we downloaded fastq files from SRA (Accession No. SRP253951). We employed Fastp [10] to obtain clean reads and then Fastv (https://github.com/OpenGene/fastv) to extract viral reads. Viral reads were aligned against the SARS-CoV-2 reference genome (NC_045512.2) with BWA MEM [33]. For each BAM file, we calculated the RNA editing levels at the 144 editing candidate sites (For “A” site: #G/depth; For “T” site: #C/depth), and then the average values for sites with sufficient coverage (≥×10) were compared to assess the editing activity difference between ADAR1-high (control) and -low (drug-treated) groups. A two-tailed Student’s t-test was used to assess the statistical significance between the two groups. We also compared the editing-level change per site upon the treatment and a binomial test with a success rate of 0.5 to test whether significantly more RNA editing sites were inhibited.

### RNA editing site clustering analysis

For each A-to-I editing site, we calculated the shortest distance (nt) between this site and its two immediate neighbor editing sites, and we obtained the median value across all the sites. We performed the same analysis for 1,000 control sets, each consisting of the same numbers of A and T, randomly sampled from the SARS-CoV-2 genome. We compared the median values of the true RNA editing set against those control sets to assess the statistical significance.

### Prediction of SARS-CoV-2 RNA secondary structure propensity

The secondary structure propensity score of the SARS-CoV-2 sequence was based on the SHAPE-MaP profiling data [36]. To consider the genomic context of the flanking sequences to compute a smoothed RNA secondary structure propensity score for each position, we averaged the scores of the up- and down-stream 170 nucleotides.

### Phylogenetic tree construction

To reconstruct phylogenetic trees, we first inferred the genome sequences of the seven samples by replacing the reference nucleotides with SNVs with a VAF of ≥ 50%. We reconstructed the phylogenetic trees using Unweighted Pair Group Method with Arithmetic mean algorithm (UPGMA) from MEGA-X [37] under two conditions: i) excluding the 10 RNA editing sites with an editing level of ≥ 30% in any sample, and ii) considering edited alleles (G or C) at these sites in the corresponding samples. We performed a bootstrapping analysis 1,000 times to evaluate the topology robustness.

### Epitope prediction

To evaluate the possibility of epitopes introduced by RNA editing, we extracted both wild-type and edited peptide sequences around the missense, high-confidence A-to-I RNA editing events. We performed eluted ligand likelihood prediction using the netMHCpan (v4.0) webserver [38]. We only considered the 100 most common HLA haplotypes across 21 populations [39].

## Supporting information

S1 Fig Depth and SNV distributions of 19 samples across the SARS-CoV-2 genome. (TIFF)

S2 Fig The distribution of 12 SNV types in different samples. (TIF)

S3 Fig Differential expression of ADAR1 isoforms upon ruxolitinib treatment. (TIF)

S1 Table Summary of metatranscriptomic datasets used in this study. (XLSX)

S2 Table Information about A-to-I RNA editing candidate sites detected in this study. Sheet 1: Annotation of 144 high-confidence RNA editing sites; Sheet 2: The numbers of the high-confidence editing sites and related edited reads in each sample; Sheet 3: Details of all potential A-to-I RNA editing (A>G/T>C) calls in each sample; and Sheet 4: Details of all SNVs types (including A>G/T>C) in each sample.
(XLSX)

S3 Table List of potential epitopes introduced by high-confidence A-to-I RNA editing candidates. (XLSX)
