# Supplementary figures and images for: RNA editing increases the nucleotide diversity of SARS-CoV-2 in human host cells
Source: PLoS Genet. 2022 Mar 30;18(3):e1010130. doi: 10.1371/journal.pgen.1010130 (PMC9000099; doi:10.1371/journal.pgen.1010130)

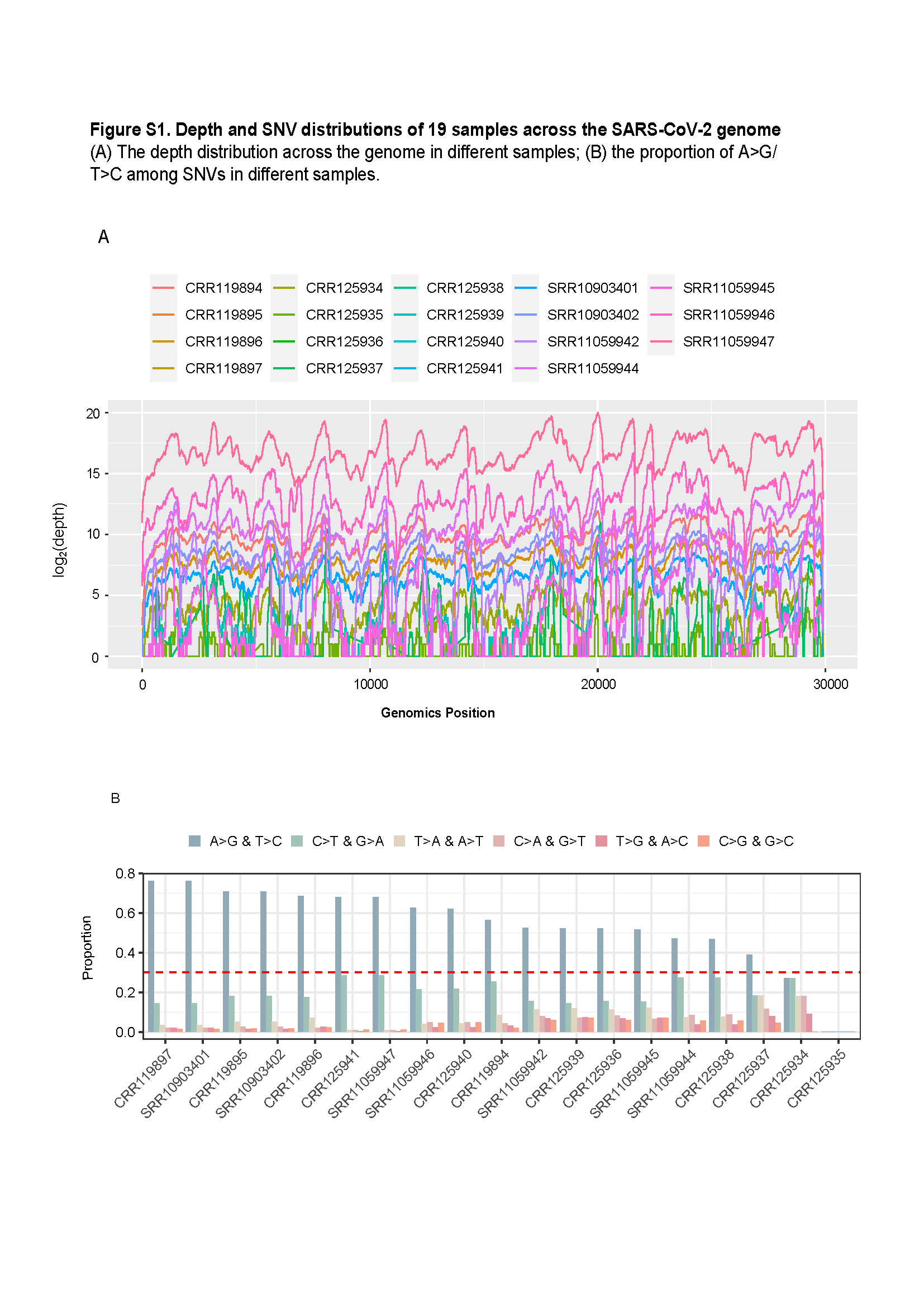

Supplement: S1 Fig — (TIFF) [file pgen.1010130.s001.tiff]

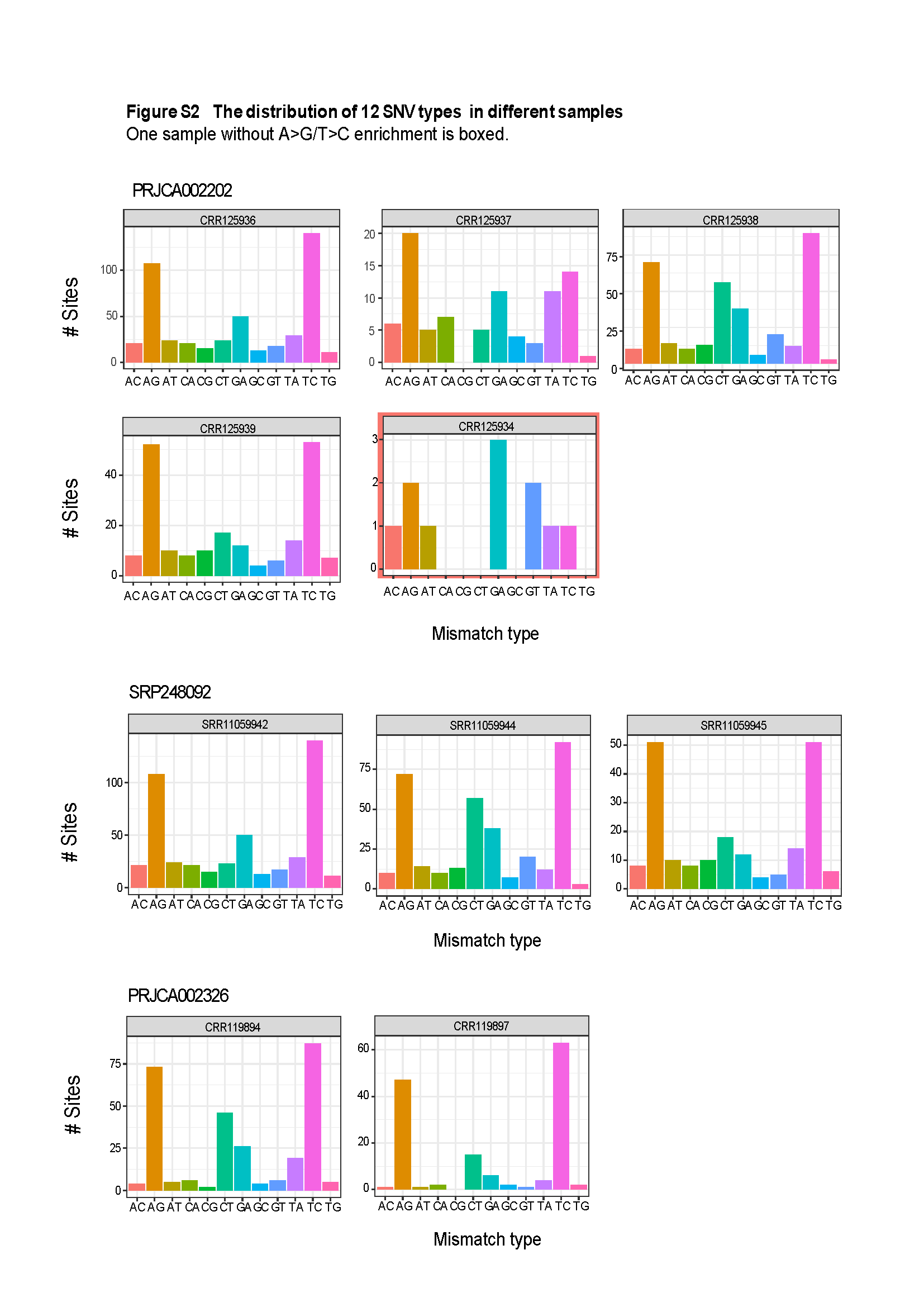

Supplement: S2 Fig — (TIF) [file pgen.1010130.s002.tif]

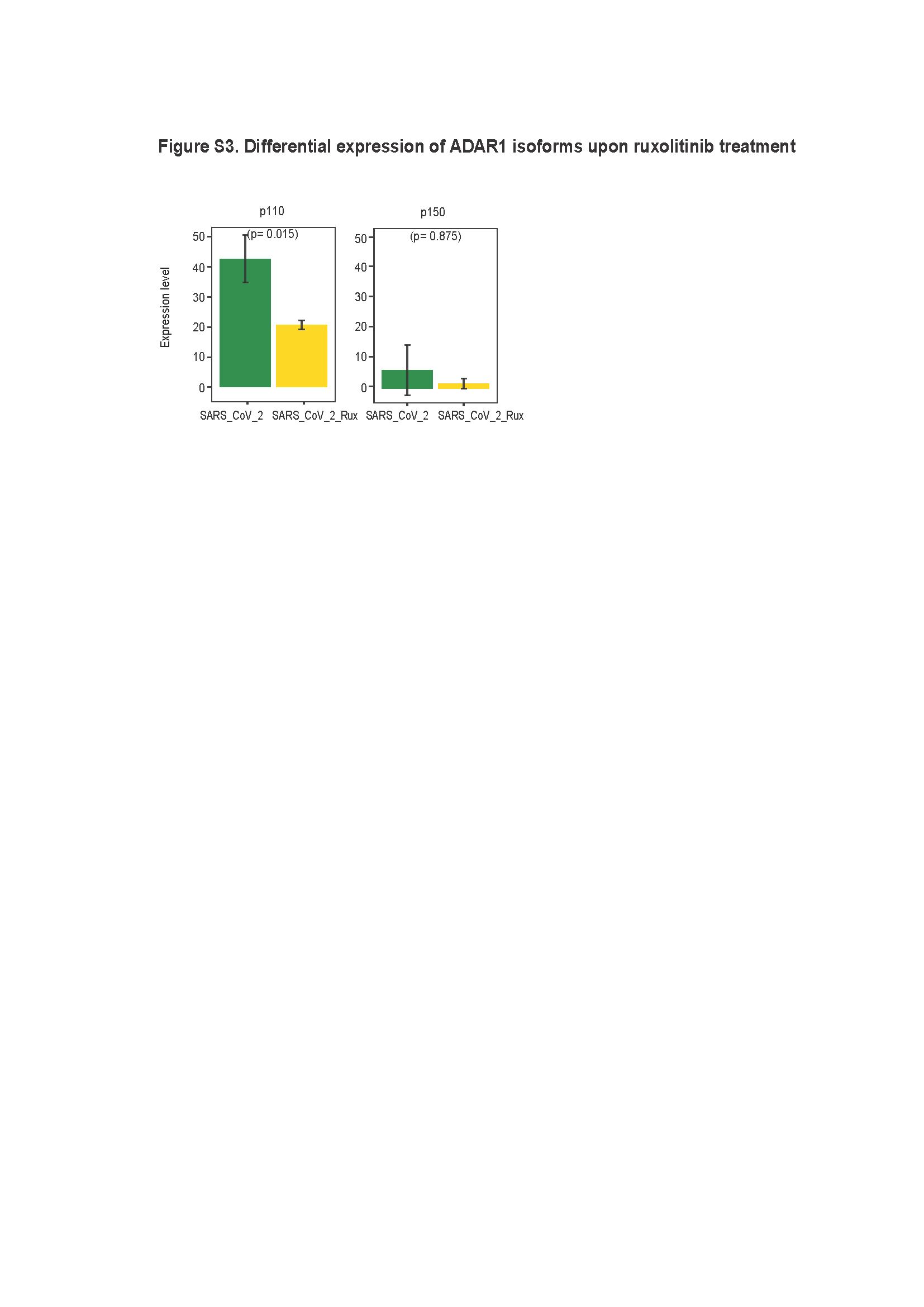

Supplement: S3 Fig — (TIF) [file pgen.1010130.s003.tif]
